# Ubiquitination and Ubiquitin-Like Modifications in Multiple Myeloma: Biology and Therapy

**DOI:** 10.3390/cancers12123764

**Published:** 2020-12-14

**Authors:** Matthias Wirth, Markus Schick, Ulrich Keller, Jan Krönke

**Affiliations:** 1 Department of Hematology, Oncology and Tumor Immunology, Charité-Universitätsmedizin Berlin, Campus Benjamin Franklin, 12203 Berlin, Germany; matthias.wirth@charite.de (M.W.); markus.schick@charite.de (M.S.); ulrich.keller@charite.de (U.K.); 2 German Cancer Research Center (DKFZ), German Cancer Consortium (DKTK), 69120 Heidelberg, Germany; 3 Max-Delbrück Center for Molecular Medicine, 13092 Berlin, Germany

**Keywords:** multiple myeloma, SUMO, NEDD, ubiquitin, PROTAC, proteasome, IMiD

## Abstract

**Simple Summary:**

Multiple myeloma is a cancer of plasma cells causing bone fractures, anemia, renal insufficiency and hypercalcemia. Despite the introduction of new drugs in the past years, it still remains incurable and most patients die from the disease. Multiple myeloma cells are characterized by the production of high amounts of monoclonal antibodies. Therefore, maintaining protein homeostasis from synthesis through folding to degradation is crucial for multiple myeloma cells. While protein ubiquitination and organized degradation are typically considered critical for cellular health, an emerging strategy is to block these processes to induce cell death in disease-state cells characterized by protein over-production. Recent development of compounds that alter the ubiquitin proteasome pathway and drugs that affect ubiquitin-like modifications appear promising in both preclinically and in clinical trials. This review summarizes the impact of protein modifications such as ubiquitination and ubiquitin-like modifications in the biology of multiple myeloma and how it can be exploited to develop new effective therapies for multiple myeloma.

**Abstract:**

Multiple myeloma is a genetically heterogeneous plasma cell malignancy characterized by organ damage and a massive production of (in-)complete monoclonal antibodies. Coping with protein homeostasis and post-translational regulation is therefore essential for multiple myeloma cells to survive. Furthermore, post-translational modifications such as ubiquitination and SUMOylation play key roles in essential pathways in multiple myeloma, including NFκB signaling, epigenetic regulation, as well as DNA damage repair. Drugs modulating the ubiquitin–proteasome system, such as proteasome inhibitors and thalidomide analogs, are approved and highly effective drugs in multiple myeloma. In this review, we focus on ubiquitin and ubiquitin-like modifications in the biology and current developments of new treatments for multiple myeloma.

## 1. Introduction

Multiple myeloma (MM) is a mature B cell neoplasm characterized by monoclonal plasma cell (PC) proliferation in the bone marrow [1]. Although new treatment modalities have prolonged the median survival time to more than 10 years in patients eligible for intensive therapy, MM is still considered incurable, and most patients die from the disease [1]. The transformed PCs produce abnormal monoclonal antibodies (M proteins) and a corresponding light chain, which may be present as Bence Jones protein in the urine. The clinical manifestations include osteolysis and pathologic fractures, hypercalcemia, renal impairment, and hematopoietic insufficiency. While multiple myeloma patients often present with these symptoms that require immediate treatment, a fraction of patients have asymptomatic smoldering myeloma—an intermediate stage of the disease between MGUS (monoclonal gammopathy of undetermined significance) and active myeloma—which can have a long asymptomatic course [1]. MM is characterized by a high degree of inter- and intra-individual genetic heterogeneity with a large number of different cytogenetic and molecular aberrations [2,3]. Chromosomal copy number alterations and translocations are considered as initiating events in MM that can also be detected in MGUS [4]. Approximately 40% of MM have a hyperdiploid karyotype that is characterized by the presence of trisomies in multiple chromosomes, usually affecting the odd chromosomes 3, 5, 7, 9, 11, 15, 19, and 21. The remaining patients carry translocations that fuse a gene to the *immunoglobulin heavy chain* (*IgH*) gene with the most frequent being t(4;14)/*IgH-MMSET*/*FGFR3*, t(14;16)*/IgH-MAF*, and t(14;20)/*IgH-MAFB* [5,6,7]. Secondary cytogenetic abnormalities that occur during the course of the disease include *IgH-MYC* translocations, chromosomal deletions like del(1p), del(17p13) comprising *TP53*, del(13q) comprising *RB1*, and amplification of 1q21. Besides chromosomal changes, recurrent gene mutations are found in MM. Gene mutations in the MAPK pathway (*NRAS, KRAS, BRAF*) are common and affect 40 to 50% of all patients. Less frequently, mutations are found in DNA damage repair genes (*TP53, ATM*), cell cycle genes (*RB1*), and transcription factors important for plasma cell differentiation (*IRF4, PRDM1, IKZF3*) [8,9]. *FAM46C* and *DIS3* are among the most frequent gene mutations in MM, but the exact biologic impact has not been fully determined [10,11,12,13,14,15,16]. Loss of the deubiquitinating enzyme CYLD, which acts as a negative regulator of nuclear factor kappa-light-chain-enhancer of activated B cells (NFκB) and Wnt-Signaling, increases the aggressiveness of MM [17]. *BIRC2*/*BIRC3* encode ubiquitin ligases involved in apoptosis regulation, and genetic deletions in multiple myeloma lead to NFκB activation [18,19]. The importance of the NFκB pathway in multiple myeloma is further highlighted by genetic or epigenetic alterations found in other genes in this pathway, such as *NIK, TRAF3, CD40, NFKB1*, or *NFKB2* [19].

Beyond genetic alterations, MM is characterized by epigenetic changes, such as aberrant DNA and histone methylation patterns [20,21,22,23]. Members of the nucleosome remodeling and deacetylase complex contribute to the regulation of DNA and histone methylation, histone acetylation, and chromatin remodeling, which play important roles in MM [22,24]. Of note, epigenetic modifiers like the histone methyltransferase *MLL*, the histone demethylase *KDM6A*, and the histone acetyltransferase *EP300* or *CHD2* and *CHD4* are mutated in MM and might contribute to the observed epigenetic changes. Understanding these mechanisms is vital, as epigenetic mechanisms affect the phenotype, clonal heterogeneity, and plasticity in MM [25]. For example, a high degree of DNA methylation and histone acetylation correlated with an aggressive immature phenotype in a syngeneic immunocompetent murine 5T33 MM model [26]. Moreover, aberrant DNA methylation patterns are a defining characteristic of MM, and there are qualitative epigenetic differences between premalignant MGUS, in which demethylation occurs primarily in CpG islets, and active myeloma, in which differentially methylated loci occur in predominantly non-CpG islets [27]. Accordingly, the de novo DNA methyltransferase DNMT3A is suppressed in MM, and low expression is associated with adverse prognosis in MM [27].

A hallmark of MM cells is the production of high amounts of monoclonal antibody. Therefore, maintaining protein homeostasis from synthesis through folding to degradation is crucial for multiple myeloma cells [28]. Under normal conditions, misfolded proteins degrade within minutes, and, if not removed early, can dramatically increase basal proteasome loading and cellular stress [29]. This proteotoxic stress can be further increased by chromosomal hyperdiploidy and MYC activation, both leading to an increased expression of many proteins, which induces an increased protein load in the cell [30].

Beyond transcriptional mechanisms, the abundance and function of proteins is controlled by highly dynamic and largely reversible post-translational modifications (PTMs). The diverse group of PTMs comprises acetylation, phosphorylation, methylation, ubiquitination, SUMOylation, and NEDDylation, which affect virtually all cellular processes [31].

Among the proteins whose function is highly regulated by PTMs are histones with more than 500 different PTMs identified [32,33,34,35]. These modifications not only regulate the chromatin structure, but also recruit corresponding enzymes that use the energy obtained from the hydrolysis of ATP to reposition nucleosomes and also induce the recruitment of proteins and complexes with specific enzymatic activities [36]. PTMs of transcription factors can be crucial for their activity and DNA binding specificity. The interplay of histone modifications, epigenetic regulators, and transcription factors lead to dysregulation of gene expression, causing aberrant regulation of oncogenes and tumor suppressors. Dissection of this interdependence might allow therapeutic targeting of these networks.

Whereas ubiquitination and the organized degradation of proteins is typically thought of as being critical to cellular health, an emerging strategy is to block those processes in order to induce cell death in disease state cells that are characterized by the over-production of proteins.

## 2. Aberrant Ubiquitination in MM

**Figure 1:**
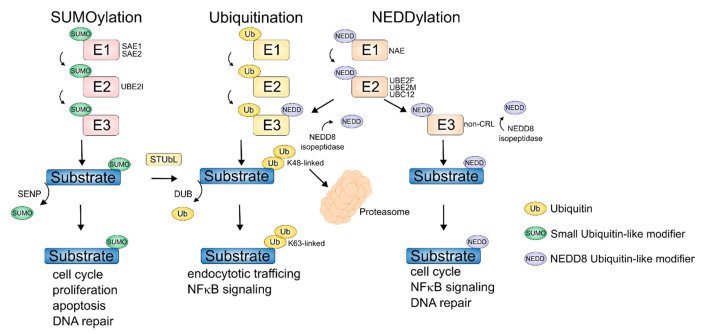
Ubiquitination, SUMOylation, and NEDDylation pathways. Conjugation of ubiquitin, small ubiquitin-like modifier (SUMO), and NEDD8 ubiquitin-like modifier through an enzymatic cascade of E1, E2, and E3 ligases to protein substrates. Crosstalk of the SUMO pathway via SUMO targeted ubiquitin ligases (STUbL) to ubiquitinate SUMOylated proteins. K48 polyubiquitinated protein substrates are degraded via the proteasome. Other lysine linkages such as K63 polyubiquitination modify the activity of a protein. Most prominent targets of NEDDylation are cullin-RING ubiquitin ligases (CRL). Both NEDDylated CRL and non-CRLs are deNEDDylated by NEDD8 isopeptidases. Deubiquitination of protein substrates is mediated via deubiquitinases (DUB). SUMOylated protein substrates are deSUMOylated via sentrin/SUMO-specific proteases (SENP). A section of the altered protein function due to PTMs shown is summarized under corresponding substrates.

The ubiquitin-proteasome system (UPS) plays an important role in the regulation of protein stability and function [37]. The dynamic process of ubiquitination keeps protein functional states in a homeostatic equilibrium (Figure 1). The conjugation of ubiquitin to target proteins takes place via an enzymatic cascade consisting of ubiquitin-activating enzymes (E1), ubiquitin-conjugating enzymes (E2), and ubiquitin-protein ligases (E3) [38]. The E3 ubiquitin ligases are the final effectors of this cascade that transfer ubiquitin to their substrates in a highly specific fashion. The specificity is determined by non-covalent binding of the so-called substrate adaptor of the E3 ligase and the degron sequence of the substrate protein. The E3 ligase covalently links ubiquitin to a lysine residue in the protein leading to monoubiquitination or polyubiquitination by linking multiple ubiquitin molecules with each other to chains. Different types of polyubiquitination exist that depend on the lysine (K) residue within ubiquitin that is used for attaching the next ubiquitin molecule, namely K6, K11, K27, K29, K33, K48, K63, or methionin at the *N*-terminus. The best characterized type of polyubiquitination is K48-linked, which marks protein substrates for proteasomal degradation by the ATP-dependent 26 S-proteasome complex [38]. K63-linked chains are involved in processes such as endocytotic trafficking, inflammation, and DNA damage repair. K11-bound polyubiquitin chains are involved in mitotic regulation and endoplasmic reticulum-associated degradation [39]. Monoubiquitination leads to a change in protein function or protein translocation.

The NFκB pathway, which is frequently altered in MM, is highly regulated by ubiquitination [40]. In histones H2A and H2B, monoubiquitination induced by E3-ligases of the polycomb repression complex 1, such as BMI1 (B cell-specific Moloney murine leukemia virus integration site 1), RING1A/RING1B (Really Interesting New Gene 1A/1B), and PCGF2 (Polycomb group RING finger protein 2), as well as by further E3-ligases such as RNF8, and 2A-HUB/hRUL138 lead to the repression of gene expression by inhibition of the RNA polymerase 2 elongation process [41,42,43,44]. In MM, increased expression of BMI1 is observed compared to normal PCs [45,46]. Furthermore, it was shown experimentally that high expression of BMI1 leads to enhanced growth of MM cells both *in vitro* and *in vivo* [47]. Ubiquitination of BMI1 is mediated by the E3 ligase consisting of speckle-type broad-complex, tramtrack and Bric a brac/poxvirus and zinc finger (BTB/POZ) protein (SPOP) and cullin 3, which also ubiquitinates the core histone mH2 A.1 [48]. This leads to a replacement of conventional H2 A and as a consequence to transcriptional repression [48,49,50]. Other SPOP substrates are involved in epigenetic regulation, such as the histone lysine methyltransferase SET domain containing 2 (SETD2) [51], the histone deacetylases HDAC6 [52], sirtuin 2 (SIRT2) [53], and the bromodomain and extra-terminal motif (BET) proteins BRD2, BRD3, and BRD4 [54,55,56]. Gain of SPOP function causes enhanced polyubiquitination and subsequent degradation of BET proteins in endometrial cancer cells [54], arguing for a tumor suppressive role for SPOP. In HEK293 cells, SPOP overexpression results in the activation of the c-Jun N-terminal kinase (JNK) pathway, and up to 99% of renal cancer patients show an increased expression of SPOP [57], whereas in MM its expression is rather heterogeneous and its role in MM needs to be evaluated [58]. SETD2 is a trimethylase that induces trimethylation of lysine 36 of the histone H3 (H3 K36 me3) [59,60]. It is involved in DNA double strand repair [61] and DNA mismatch repair [62] and is mutated especially in relapsed MM patients [63,64].

### Exploiting the Ubiquitin Proteasome System Therapeutically

**Figure 2:**
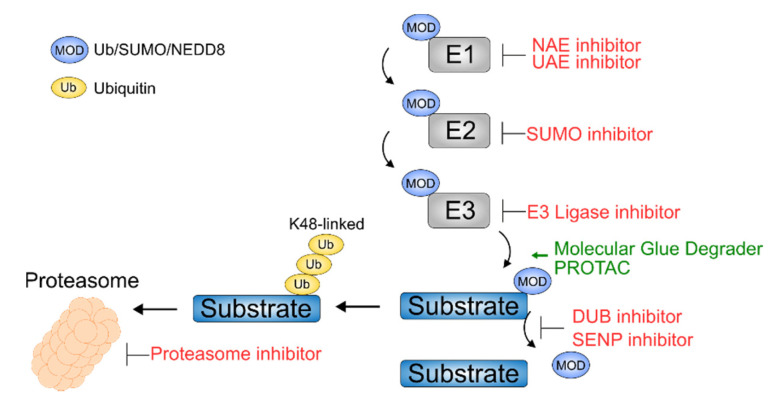
Exploiting ubiquitination, SUMOylation, and NEDDylation therapeutically. Displayed are inhibitors of E1 enzymes such as the NEDD8 activating enzyme 1 (NAE) and ubiquitin activating enzyme 1 (UAE), inhibitors of the E2 enzyme UBC9/UBE2I, E3 ligase inhibitors, deubiquitination (DUB) inhibitors, sentrin/SUMO-specific protease (SENP) inhibitors, and proteasome inhibitors. In addition, a new class of therapeutics is shown, such as molecular glue degraders and proteolysis targeting chimeras (PROTAC), which use the cellular intrinsic degradation machinery to degrade specific substrate proteins.

The ubiquitin–proteasome system provides many opportunities for pharmacologic intervention by modulating the function of ubiquitinating enzymes, deubiquitinating enzymes (DUBs), and the proteasome. The highly potent ubiquitin activating enzyme 1 (UAE1) inhibitor TAK-243 blocks the ubiquitin conjugation cascade (Figure 2), resulting in proteotoxic stress and MM cell death [65] recently entered in clinical trials (Table 1). Proteasome inhibitors and thalidomide analogs, also known as immunomodulatory drugs (IMiDs), are highly active and approved for MM and are the prime example for therapies targeting the UPS in cancer. Proteasome inhibitors (PI) inhibit the proteasomal degradation of ubiquitinated proteins (Figure 2). This leads to a massive accumulation of proteins, an associated unfolded protein response (UPR), and finally to the induction of the intracellular apoptosis machinery leading to tumor cell death [66,67,68]. MM cells are characterized by a high synthesis rate of immunoglobulins, aneuploidy, gene mutations, and genomic instability, which elevates proteotoxic stress by an accumulation of polyubiquitinated proteins and an induction of the UPR [69]. As a result, MM cells are particularly dependent on proteostasis signaling pathways and therefore are highly sensitive to proteasome inhibition [69]. Inhibition of the proteasome further leads to increased abundance of certain tumor suppressors like IκB, which leads to impaired NFκB activity and an interference of anti-apoptotic signaling in MM cells [70,71]. Furthermore, several studies have shown that sensitivity towards proteasome inhibition correlates with dependence of the transcription factor MYC, which is essential for MM [72]. Additionally, bortezomib alters homologous recombination by abrogating histone H1 K63 polyubiquitination, which impairs the recruitment of BRCA1 and RAD51 [73]. In 2003, bortezomib became the first Food and Drug Administration (FDA) approved PI for the treatment of refractory MM (Table 1) [74,75]. New generations of PIs were developed that are more active and have a more favorable toxicity profile, especially lower rates of peripheral neuropathies, which are one of the major reasons leading to discontinuation of bortezomib therapy (Table 1) [76]. In 2012, the irreversibly binding and selective PI carfilzomib was approved by the FDA for the treatment of relapsed or refractory MM [77]. In the phase 3 ENDEAVOR trial, carfilzomib significantly prolonged progression-free survival and overall survival as compared to bortezomib in relapsed MM patients [78]. In contrast to bortezomib, carfilzomib irreversibly and selectively inhibits the 5 subunit of the 20 S proteasome, which harbors chymotrypsin-like activity; additionally, it inhibits the low-molecular mass protein-7 (LMP7) of the immunoproteasome [79].

Thalidomide and its analogs, lenalidomide and pomalidomide, are immunomodulatory drugs (IMiDs) that are approved for the treatment of multiple myeloma [93]. Lenalidomide, but not the other IMiDs, is also active in patients with myelodysplastic syndrome (MDS) with deletion of chromosome 5 q (del(5 q)) [94]. Thalidomide (brand name “contergan”) was initially developed as a sedative in the 1950s but a few years later became notorious for causing teratogenicity with severe limb deformation (phocomelia) in more than 10,000 children during the so-called contergan catastrophe. Ito et al. identified in 2010 cereblon (CRBN), which is the substrate adaptor of the CRBN-CRL4 E3 ubiquitin ligase, as the primary target of all IMiDs responsible for the teratogenicity as well as the anti-cancer effects [95]. Thalidomide and its analogs act as molecular glue degraders (Figure 2) that modulate the substrate specificity of the CRBN-CRL4 E3 ubiquitin ligase to induce ubiquitination and degradation of so-called neo-substrates that include SALL4, CK1α, and the lymphoid transcription factors ikaros (IKZF1) and aiolos (IKZF3) [96,97,98,99,100,101]. IKZF1 and IKZF3 are key regulators of B-cell differentiation and are highly expressed in MM and other mature B-cell lymphomas [102]. Degradation of IKZF1 and IKZF3 leads to transcriptional downregulation of IRF4 and MYC, two essential transcription factors in MM, and thereby inhibits proliferation of MM cells. IKZF1 and IKZF3 degradation provides the basis for the selective activity of IMiDs in MM and some lymphomas that depend on these transcription factors. Depletion of IKZF1 and IKZF3 further induces interleukin-2 release from T cells, explaining another major property of IMiDs [96,98,99,103]. IKZF1 and IKZF3 are commonly targeted by the approved thalidomide, lenalidomide, and pomalidomide as well as the new analogs iberdomide and avadomide that are currently being tested in clinical trials. Chemical modifications in the IMiD molecular structure alters their substrate specificity. One example is lenalidomide, which, unlike other IMiD analogs, targets casein kinase 1 isoform alpha (CK1α) in del(5 q) MDS [97]. CC-885 and CC-90009 are new thalidomide analogs that induce degradation of the translational termination factor GSPT1 besides IKZF1 and have activity in a much broader spectrum of cancer types but are also predicted to be more toxic to normal cells [82].

While the concept of molecular glue degraders as cancer therapies was firstly described for thalidomide analogs, recently additional drugs acting through this mechanism were found. The sulfonamide indisulam that is being tested in clinical trials for cancer induces ubiquitination and degradation of the splicing factor RBM39 via the DCAF15-CRL4 E3 ubiquitin ligase [104]. The preclinical small molecule CR8 acts as a molecular glue degrader that indirectly recruits cyclin K through CDK12 to the DDB1-CUL4 E3 ligase [83]. These examples demonstrate that induction of ubiquitin ligase mediated degradation provides a promising drug mechanism that can be exploited to effectively target proteins including those previously considered “undruggable”, such as transcription factors.

The spectrum of targetable proteins was highly extended through the development of proteolysis-targeting chimeras (PROTACs), which are bi-functional molecules. Here, the E3 ligase-binding moiety (e.g., thalidomide for CRBN) is linked to another small molecule that binds a protein of interest [84]. This technique was used to generate different molecular degraders such as dBet1 [84] and ARV-825 [85], which link a derivative of JQ1 and OTX015, respectively, to a thalidomide residue and thus can degrade the bromodomain protein BRD4 through the CRBN E3 ligase. A large spectrum of proteins has been successfully shown to be degraded by rationally designed PROTACs, including kinases (e.g., CDK4/6, receptor tyrosine kinases) [105,106,107,108], epigenetic regulators (e.g., BRDs, HDACs) [84,85,109,110], hormone receptors (androgen and estrogen receptor) [111], ubiquitin ligases (VHL, CRBN) [112,113], and KRAS [114]. Many of these PROTACs were shown to be effective *in vitro* and in mouse cancer models. In 2020, ARV-110, which degrades the androgen receptor, was the first PROTAC to reach phase 1 clinical trials (Table 1). ARV-110 showed clinical activity in prostate cancer, and maybe even more important for the future clinical development of PROTACs, was well tolerated with low toxicity. Protein degraders such as molecular glues and PROTACs therefore open up a completely new field for the development of new-targeted therapeutics in cancer. Besides degradation of the target proteins, many IMiD-based PROTACs retain their activity on IKZF1 and IKZF3, which may be an unwanted effect in several applications. In contrast, in MM this may further enhance the activity of such PROTACs by intramolecular synergy by targeting the protein of interest together with IKZF1 and IKZF3.

## 3. Deubiquitinating Enzymes (DUBs)

Protein ubiquitination is a highly dynamic and reversible process. Ubiquitination of a substrate through a ubiquitin ligase can be reversed by counteracting DUBs. Deubiquitination of a protein prevents proteasomal degradation and leads to protein stabilization (Figure 1) [115,116]. However, some DUBs, such as USP14, are themselves associated with the proteasome and enhance its activity [117]. So far, about 100 DUBs have been identified in human cells, but for most of them the substrate proteins and exact functions have not been well characterized [118]. There are at least five subclasses of DUBs: (1) ubiquitin-specific proteases (USP) that contain two conserved Cys- and His-box motifs; (2) ubiquitin-carboxyterminal hydrolases (UCH), which hydrolyze amides and esters at the C-terminus of the ubiquitin; (3) ovarian tumor (OUT)-related proteases containing conserved Cys, His, and Asp residues; (4) ataxin-3, the only proven member of its class so far, characterized by a Josephin domain, which contains few similarities with the Cys and His boxes of the first two classes; and (5) proteasome subunit DUBs, such as Rpn11/POH1, which has characteristics of a metalloprotease specific for protein-bound ubiquitin [116,119].

Several DUBs have been implicated in histone deubiquitination, including USP3, USP12, USP22, and USP46, which deubiquitinate both histones H2A and histones H2B [120]. Histone H2A-specific DUBs include 2A-DUB/MYSM1, USP16, USP21, and BRCA1-associated protein-1 (BAP1) [120]. Histone H2B-specific DUBs are UBP8 and UBP10 [120].

Histone H2A DUB 2A-DUB/MYSM1 has been associated with hematopoiesis and lymphoid differentiation [121,122,123]. Myb Like, SWIRM And MPN Domains 1 (*Mysm1*) knockout mice exhibit a phenotype with a severe bone marrow (BM) hematopoietic defect, which leads to the development of lymphopenia, anemia, and thrombocytosis [124]. Due to the functionally damaged hematopoietic stem cell population, MYSM1 plays an important role in the maintenance of the hematopoietic stem cell population (HSC). In addition, another study confirmed that the deletion MYSM1 restores dormant HSCs to a cyclic state and enhances apoptosis of HSCs, leading to depletion in the stem cell pool through blocked recruitment of the transcription factors GATA2 and RUNX1 to the *GFI1* locus [124]. Homozygous missense mutation of MYSM1, which is the catalytic site within the DUB (JAMM)/MPN domain, has been associated with complete absence of B lymphocytes, T cell lymphopenia, defective hematopoiesis, and developmental abnormalities, which further supports the essential role of MYSM1 in human hematopoietic development [125]. 

The deubiquitinase USP7 stabilizes MAF proteins, protects MDM2 (the ubiquitin ligase for TP53) from degradation, increases transcriptional activity, and promotes MM cell proliferation [126,127]. In *Drosophila*, it has been shown that USP7 contributes to epigenetic silencing of homeotic genes by polycomb and selectively deubiquitinates histone H2B [128]. Furthermore, the transcriptional regulator 1 (ASXL1), which is essential for myeloid differentiation, is stabilized by USP7 in acute myeloid leukemia (AML) [129]. Besides, USP7 targets the proteins of the FOXO family in lung cancer and DNMT1 in colon cancer and acts as a deubiquitinase for SUMO (small ubiquitin like modifier) [130].

### Therapeutic Targeting of DUBs

Since altered DUB activity is associated with many cancer-associated processes, and DUB inhibition provides another opportunity for targeting currently undruggable proteins, extensive efforts are being made to develop potent and specific DUB inhibitors.

Targeting of USP7 by the inhibitor P5091 induces cellular apoptosis of cancer cells in MM and counteracts bortezomib resistance [87]. Recently, more potent and selective USP7 inhibitors, including FT671, GNE6776, and XL-188, were described with superior activity in MM and other cancer types [88,89,90]. By inhibiting USP7 function, the proteins murine double minute 2 homolog (MDM2) and murine double minute X (MDMX), which are the main negative regulators of the tumor suppressor TP53, are destabilized, thus enhancing TP53 levels and activating apoptosis. Preclinical studies have shown promising results *in vitro* and *in vivo* in several cancer models including MM (Table 1) [88,89,90].

The deubiquitinating enzymes USP14 and UCHL5 are highly expressed in MM cells and are involved in MM tumorigenesis [131]. Global analysis of the proteome and ubiquitinome identified the histone acetyltransferase HAT1 and the linker histone H1.4 as USP14 targets [132]. IU1, identified as a small molecule inhibitor of USP14, has been shown to reduce chain trimming and stimulate proteasome degradation [133] and enhances proteasome activity [134]. The USP14-specific inhibitor VLX1570 showed similar effects and resulted in decreased cell growth and apoptosis induction of MM cells [91].

In a recent study, DUB3 was shown to promote BRD4 deubiquitination and stabilization in various cancer cell lines [135]. Since increased abundance of the BET protein BRD4 is a key factor conferring resistance to BET inhibitors [56], targeting DUB3 may provide an effective way to overcome this.

## 4. NEDDylation

The ubiquitin-like protein neural-precursor-cell-expressed developmentally down-regulated 8 (NEDD8) is covalently bound to proteins through NEDDylation. NEDDylation occurs via an isopeptide bond at the carboxyl group of the C-terminal glycine of NEDD8 to the ε amino group of a lysine in the target protein (Figure 1). Although each ubiquitin-like pathway exhibits similar structural and mechanistic features of the ubiquitin pathway (E1–E3 cascades), the biological consequences are different, so that each pathway is ultimately associated with different functions. For example, the NEDD8 signaling pathway plays a crucial role in the activation of the ubiquitin-E3 ligase activity of cullin-RING E3 ligases (CRL) [136]. NEDD8 binds different cullin elements, and the RING-activated ubiquitin-bound ubiquitin-conjugating enzyme E2 D4 (UBE2D). Here, NEDD8 acts as a nexus that causes the E3 molecular machine to transfer the ubiquitin to its target protein, such as phosphorylated inhibitor of nuclear factor kappa B alpha (IκBα) [137]. The classical NFκB signaling pathway depends on the activation of the IκB kinase (IKK) complex, which leads to a phosphorylation of IκB and subsequent K48-ubiquitination of phospho-IκB by the CRL E3-ligase β-transducin-repeat-containing protein (βTrCP) and finally to its proteasomal degradation [138]. NEDDylated βTrCP leads to the transfer of ubiquitin from the E2-ubiquitin-conjugating enzyme UBE2D to the phosphorylated IκBα substrate [137]. Targeting of this pathway is considered very promising, since both the classical and the alternative NFκB pathway, are frequently activated in MM [139], [140]. NEDDylation is also evident in histone modifications [34,141]. The RING E3 ligase RNF111 leads to increased NEDDylation of histone H4 and RNF168 leads to decreased histone H2 A NEDDylation. This results in H2A ubiquitination and the recruitment of phospho-H2A.X, which is part of the DNA damage response [34,141]. Impaired homeostasis of genes such as *BRCA1*, *CHEK1*, *CHEK2,* which are important for the mitotic process, are associated with amplification of the centrosome, chromosome instability, and a poor prognosis in MM [142]. 

### NEDDylation as Therapeutic Target

Pevonedistat (MLN-4924) is a selective inhibitor of the NEDD8-activating enzyme (NAE) and thus inhibits neddylation of proteins. Many cullin-related ubiquitin ligases (CRLs) depend on neddylation, and pevonedistat thus leads to an accumulation of CRL substrates including DNA replication factor CDT1, the cyclin-dependent kinase inhibitor 1 B (p27^Kip1^), and phosphorylated IκBα [92,143]. In a phase I clinical trial, pevonedistat showed clinical activity in lymphoma and MM patients and was well tolerated (Table 1) [143]. The common effect on stabilizing proteins by NEDDylation inhibition and proteasome inhibitors provides a rational for combining these drugs in MM. Indeed, synergistic activity was observed [144] *in vitro*, and a clinical trial combining pevonedistat and ixazomib is ongoing (Table 1). In contrast to the synergy with proteasome inhibitors, NAE inhibitors abrogate the activity of CRBN-CRL4 E3 ubiquitin ligase and thus antagonize the IMiD-induced degradation of neo-substrates.

Besides CRLs, NAE inhibition blocks the recruitment of the BRCA1 DNA damage repair complex and a combination with PARP inhibition inhibited cell growth of non-small cell lung cancer (NSCLC) cells [145]. Such combination therapies may also be effective in MM but need further exploration.

## 5. SUMOylation

Covalent binding of the small ubiquitin-like modifier (SUMO) to corresponding substrates (SUMOylation) controls various biological processes including subcellular localization, function, and binding of targets to proteins [146]. There are four SUMO isoforms that are expressed in mammals and cleaved C-terminally by SUMO-specific proteases. Similar to the enzymatic cascade in ubiquitination, a complex of E1 enzymes SUMO activating enzymes 1 (SAE1) and 2 (SAE2/UBA2 (activates SUMO and transfers it to the conjugating E2 enzyme UBC9/UBE2I (Figure 1) [147,148]. By interaction of UBE2I with a corresponding target protein, the transfer of SUMO to a lysine of the target is mediated by formation of an amide bond between the lateral ε amino group of lysine and the SUMO related lateral carboxy group of aspartate or glutamate. Target selection can be enhanced by specific SUMO (E3) ligases. These act as adapters to bind UBE2 I to the target protein. The E3 ligases include members of the protein inhibitor of activated STAT (PIAS) family, the polycomb protein PC2 and the RAs-related nuclear binding protein 2 RanBP2, which is part of the nucleoporin family [147,148]. There are varieties of SUMOylation substrates involved in critical cellular processes, including cell cycle regulation, proliferation, apoptosis, drug toxicity, and DNA repair; these affect the development and progression of cancer cells and the development of drug resistance [147,148].

### SUMOylation as Therapeutic Target

The SUMOi TAK981 is a specific inhibitor of the SAE complex and blocks transfer to the conjugating E2 enzyme UBE2I, thereby inhibiting *de novo* SUMOylation [149]. In functional genetic and pharmacological experiments, a synthetic lethality of MYC and the SUMO pathway has been described [150,151,152]. A clinical phase 1 trial is currently being conducted to test the effectiveness of the SUMO inhibitor TAK981 in patients with solid tumors and hematological malignancies (Table 1). Since MM is characterized by a high UPR and is driven by MYC, MM cells may be particularly sensitive to SUMOi. Since SUMO is tightly connected to the DNA damage response pathway, and proteasome inhibition is associated with a “BRCAness” phenotype [73], a combination of PI and SUMOi may be synergistic in MM patients. SUMO targeted ubiquitin ligases (STUbLs) regulate the stability of certain SUMOylated protein substrates (Figure 1) [153]. The role of STUbLs in therapeutic response is not fully investigated. Since the STUbL ring finger protein 4 (RNF4) is required in DNA damage response (DDR) [154], also here a potential targeting by molecular glue degraders and a combination with drugs, exploiting DDR vulnerabilities, such as PARP inhibitors, might be an option for future drug development and treatment modalities.

## 6. DeSUMOylation

Like ubiquitination, the SUMOylation of substrate proteins can be reversed by so-called SUMO-specific proteases. In contrast to DUBs, only one class of deSUMOylases with six family members is reported in mammals [147,148]. This class is called the sentrin-specific protease (SENP) protein family. SENP1 is located in the nucleus and deconjugates several SUMOylated proteins [155]. *Senp1* knockout mice have erythropoietic defects in the fetal liver [156]. In MM, SENP1 is induced by IL6 and is involved in proliferation and survival of MM cells [157]. An overexpression of SENP1 in inflammatory synovial fibroblasts decreased acetylation of the MMP-1 promoter by an accumulation of HDAC4 [158]. SENP2 has a broad substrate specificity similar to SENP1 [159]. Silencing of SENP2 in MM contributes to the development of bortezomib resistance mediated by the activation of NFκB [160]. Similarly, it has been shown in other cancers that activation of NFκB may also induce resistance to therapy [160]. Targeted inhibition of NFκB activation could therefore be an important starting point for targeting MM and overcoming resistance [161]. However, it is currently unexplored whether SUMO inhibitors or rather SENP inhibitors would be possible combination partners for either PIs or IMiDs.

## 7. Conclusions

Ubiquitination and Ubiquitin-like modifications are highly dynamic posttranslational protein modifications that regulate protein homeostasis and key pathways in MM. The further development of small molecules that specifically modulate these PTMs are a highly promising approach for the development of new targeted therapies for MM and other cancers.

## Figures and Tables

**Table 1:** Drugs and compounds targeting Ub- and Ub-like pathways.

Drug/Compound	Target	Main Substrates	Development	Reference/ClinicalTrials.gov Identifier
Proteasome inhibitor				
Bortezomib, Carfilzomib, Ixazomib	Proteasome	many	Approved	[75,78,80]
Marizomib	Proteasome	many	Clinical trials	NCT03345095, NCT00461045
Oprozomib	Proteasome	many	Clinical trials	NCT02939183
Molecular glue degrader				
Thalidomide, Lenalidomide, Pomalidomide	CRBN	IKZF1/3 CK1 a SALL4	Approved	[81]
Iberdomide, Avadomide	CRBN	IKZF1/3	Clinical trials	NCT04564703, NCT04392037, NCT03283202, NCT03834623
CC-885, CC-90009	CRBN	GSPT1	Clinical trials	[82], NCT02848001
Indisulam	DCAF15	RBM39	Clinical trials	NCT01692197
CR8	DDB1	Cyclin K	Pre-clinical	[83]
PROTAC				
ARV-110	VHL	AR	Clinical trials	NCT03888612
ARV-825, dBET1	CRBN	BRD4	Pre-clinical	[84,85]
E1 ubiquitin ligase inhibitor				
TAK-243/ MLN7243	UAE1	many	Clinical trials	NCT03816319
E3 ubiquitin ligase inhibitor				
AMG-232, ALRN-6924	MDM2	TP53	Clinical trials	NCT01723020, NCT03031730, NCT03654716
SZL-P1–41	SKP2	p21^Cip1^, p27^Kip1^, p57^Kip2^	Pre-clinical	[86]
SUMOylation inhibitor				
TAK-981	UBE2 I	many	Clinical trials	NCT03648372, NCT04074330
DUB inhibitor				
P5091, FT-671, GNE-6776, XL188	USP7	MDM2, TP53	Pre-clinical	[87,88,89,90]
VLX1570	USP14	many	Pre-clinical	[91]
NEDDylation inhibitor				
Pevonedistat	NAE1	CRLs	Clinical trials	NCT03770260, NCT00722488
TAS4464	NAE1	CRLs	Pre-clinical	[92]

p21^CIP1^: cyclin-dependent kinase inhibitor 1A (CDKN1A); p27^KIP1^: cyclin-dependent kinase inhibitor 1B (CDKN1B); p57^Kip2^: cyclin-dependent kinase inhibitor 1C (CDKN1C); CRL, Cullin ring ubiquitin ligase.
